# Exome sequencing in large, multiplex bipolar disorder families from Cuba

**DOI:** 10.1371/journal.pone.0205895

**Published:** 2018-10-31

**Authors:** Anna Maaser, Andreas J. Forstner, Jana Strohmaier, Julian Hecker, Kerstin U. Ludwig, Sugirthan Sivalingam, Fabian Streit, Franziska Degenhardt, Stephanie H. Witt, Céline S. Reinbold, Anna C. Koller, Ruth Raff, Stefanie Heilmann-Heimbach, Sascha B. Fischer, Bipolar Disorder Working Group of the Psychiatric Genomics Consortium, Stefan Herms, Per Hoffmann, Holger Thiele, Peter Nürnberg, Heide Löhlein Fier, Guillermo Orozco-Díaz, Deinys Carmenate-Naranjo, Niurka Proenza-Barzaga, Georg W. J. Auburger, Till F. M. Andlauer, Sven Cichon, Beatriz Marcheco-Teruel, Ole Mors, Marcella Rietschel, Markus M. Nöthen

**Affiliations:** 1 Institute of Human Genetics, University of Bonn School of Medicine & University Hospital Bonn, Bonn, Germany; 2 Department of Genomics, Life & Brain Center, University of Bonn, Bonn, Germany; 3 Human Genomics Research Group, Department of Biomedicine, University of Basel, Basel, Switzerland; 4 Institute of Medical Genetics and Pathology, University Hospital Basel, Basel, Switzerland; 5 Department of Psychiatry (UPK), University of Basel, Basel, Switzerland; 6 Department of Genetic Epidemiology in Psychiatry, Central Institute of Mental Health, Medical Faculty Mannheim, University of Heidelberg, Mannheim, Germany; 7 Department of Biostatistics, Harvard T.H. Chan School of Public Health, Boston, Massachusetts, United States of America; 8 Institute of Neuroscience and Medicine (INM-1), Research Center Jülich, Jülich, Germany; 9 Cologne Center for Genomics, University of Cologne, Cologne, Germany; 10 Institute of Genomic Mathematics, University of Bonn, Bonn, Germany; 11 Emergency & Critical Care Unit, Coin-Guadalhorce, Málaga, Spain; 12 National Centre of Medical Genetics, Medical University of Havana, Havana, Cuba; 13 General Hospital of Bayamo, Bayamo, Cuba; 14 Experimental Neurology, Goethe University Hospital, Frankfurt, Germany; 15 Department of Translational Research in Psychiatry, Max Planck Institute of Psychiatry, Munich, Germany; 16 Department of Neurology, Klinikum rechts der Isar, Technical University of Munich, Munich, Germany; 17 Psychosis Research Unit, Aarhus University Hospital, Risskov, Denmark; Odense University Hospital, DENMARK

## Abstract

Bipolar disorder (BD) is a major psychiatric illness affecting around 1% of the global population. BD is characterized by recurrent manic and depressive episodes, and has an estimated heritability of around 70%. Research has identified the first BD susceptibility genes. However, the underlying pathways and regulatory networks remain largely unknown. Research suggests that the cumulative impact of common alleles with small effects explains only around 25–38% of the phenotypic variance for BD. A plausible hypothesis therefore is that rare, high penetrance variants may contribute to BD risk. The present study investigated the role of rare, nonsynonymous, and potentially functional variants via whole exome sequencing in 15 BD cases from two large, multiply affected families from Cuba. The high prevalence of BD in these pedigrees renders them promising in terms of the identification of genetic risk variants with large effect sizes. In addition, SNP array data were used to calculate polygenic risk scores for affected and unaffected family members. After correction for multiple testing, no significant increase in polygenic risk scores for common, BD-associated genetic variants was found in BD cases compared to healthy relatives. Exome sequencing identified a total of 17 rare and potentially damaging variants in 17 genes. The identified variants were shared by all investigated BD cases in the respective pedigree. The most promising variant was located in the gene *SERPING1* (p.L349F), which has been reported previously as a genome-wide significant risk gene for schizophrenia. The present data suggest novel candidate genes for BD susceptibility, and may facilitate the discovery of disease-relevant pathways and regulatory networks.

## Introduction

Bipolar disorder (BD) is a severe neuropsychiatric disorder with an estimated heritability of around 70% [1]. BD is characterized by recurrent episodes of depression and mania or hypomania. The lifetime prevalence of BD in the general population is around 1% [2], and the World Health Organization ranks BD among the top 10 leading causes of disease burden for the age group 15-to-44 years (The global burden of disease: 2004 update) [3, 4].

Molecular genetic research indicates that BD is a multifactorial disorder, in which both genetic and environmental factors impact disease susceptibility [5]. Recent genome-wide association studies (GWAS) have identified the first BD susceptibility genes [3, 6–10]. However, the underlying pathways and regulatory networks remain largely unknown [11]. Research has shown that the cumulative impact of common alleles with small effects explains only around 25–38% of the phenotypic variance for BD [12, 13]. A plausible hypothesis therefore is that rare, high penetrance variants may contribute to BD susceptibility. One approach to the evaluation of this hypothesis is the investigation of large, multigenerational pedigrees with a high prevalence of BD. In such pedigrees, the existence of a genetic variant of strong effect—as inherited from a common ancestor—may be more likely [14].

Results from initial whole exome and genome sequencing studies of BD suggest that the application of next generation sequencing is a promising method for the identification of rare variants in BD candidate genes and disease-related pathways. Recent investigations have comprised pedigree and trio analyses, as well as case-control studies [15–17]. Preliminary results suggest an enrichment of rare variants in gene-sets previously implicated in BD, such as mitogen-activated protein kinase (MAPK) signaling; axon guidance; calcium signaling; cyclic adenosine monophosphate response element binding protein (CREB) signaling; and potassium channels [18–24]. These results also implicate gene-sets identified in *de novo* studies of autism and schizophrenia in BD etiology [17]. A recent trio-based exome sequencing study suggested that *de novo* mutations may play a role in BD etiology, particularly in patients with early disease onset [15]. However, the overlap in implicated candidate genes between studies was limited, suggesting that the analysis of further samples and pedigrees is warranted before more definitive conclusions can be drawn.

The aim of the present study was to investigate the role of rare, nonsynonymous, and potentially functional variants in BD susceptibility by performing whole exome sequencing in two large, multiply affected BD families from Cuba. The high lifetime prevalence of BD in these pedigrees renders them promising in terms of the identification of high penetrance genetic risk variants. In addition, polygenic risk score (PRS) based analysis, that uses cumulated information contained in many small association signals throughout the genome, was performed to elucidate the underlying genetic architecture.

## Materials and methods

### Phenotypic assessment and DNA collection

The present study was performed within the context of the Cuban Danish project on BD and schizophrenia, and was approved by the ethics committee of the National Center of Medical Genetics in Havana, Cuba. Written informed consent was obtained from all participants prior to inclusion.

All participants were recruited in the southeastern region of Cuba and underwent initial psychiatric assessment in 1997 and follow-up assessment in 2016. Each participant was examined at home by a research psychiatrist in cooperation with a local psychiatrist.

Best estimate diagnoses were assigned on the basis of: the Structured Clinical Interview for the Diagnostic and Statistical Manual of Mental Disorders (DSM-IV) (SCID); structured information obtained from other family members; and information from the responsible psychiatrist. DNA was obtained from whole blood by salting-out with saturated sodium chloride solution [25].

### Sample description

**Fig 1:**
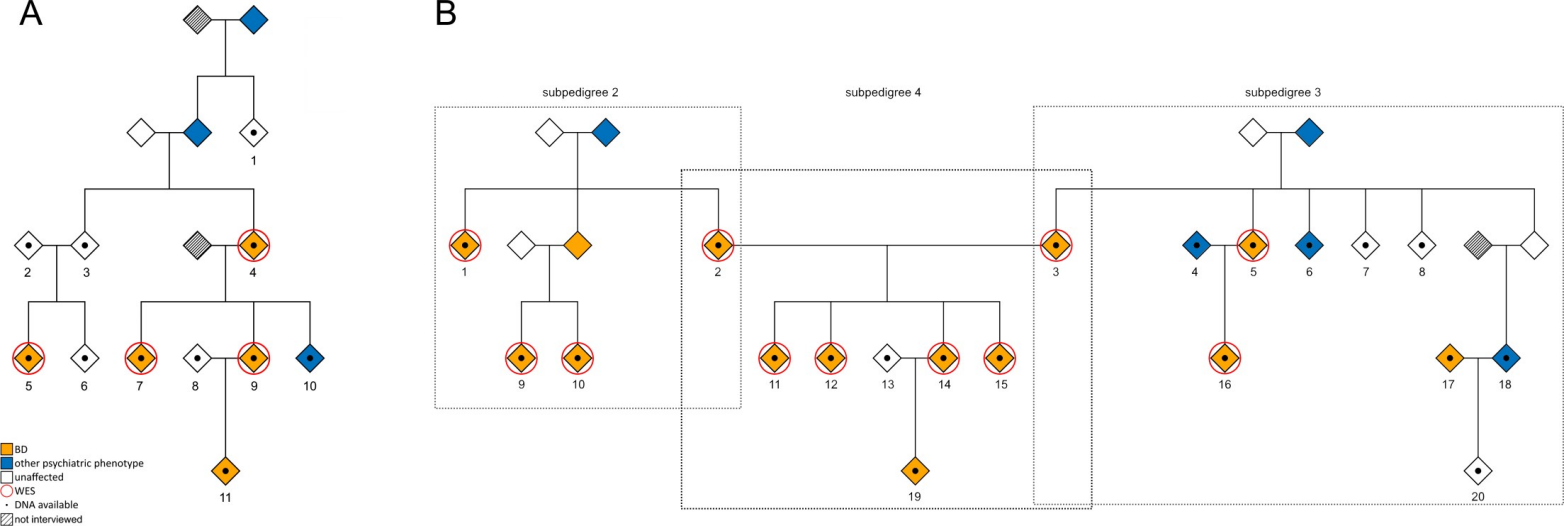
Pedigrees of the two investigated Cuban families. DNA was available for all numbered individuals depicted by a diamond with a central dot. Exome sequencing was performed on subjects framed with a red circle. Orange indicates a diagnosis of BD I, BD II, or BD NOS. Blue indicates other psychiatric phenotypes, comprising recurrent MDD, MDD single episode, and alcohol abuse. Unaffected individuals are indicated by white diamonds. To preserve the anonymity and confidentiality of the families, no information is shown concerning sex or mortality. (A) Pedigree of family 1, including four cases selected for exome sequencing. (B) Family 2 was divided in pedigrees 2, 3, and 4. A total of 11 cases from family 2 were selected for exome sequencing.

The first family comprised six generations and 32 individuals (41% male), including 17 individuals with a known psychiatric disorder. DNA was available for a total of 11 individuals (Fig 1A) with the following diagnoses: BD type I (BD I, n = 3); BD type II (BD II, n = 1); BD not otherwise specified (BD NOS, n = 1); alcohol abuse (n = 1); and no current or lifetime psychiatric disorder (healthy subjects, n = 5). The second family (Fig 1B) was bilineal. This family comprised four generations and 71 individuals (54% male), including 32 individuals with a known DSM-IV psychiatric disorder. DNA was available for 20 individuals with the following diagnoses: BD I (n = 4); BD II (n = 9); major depressive disorder (MDD, n = 1); alcohol abuse (n = 2); and healthy subjects (n = 4). To facilitate the analyses, family 2 was divided into subpedigrees 2, 3, and 4. For the 15 individuals selected for whole exome sequencing, additional clinical information is provided in S1 Table.

### Single nucleotide polymorphism (SNP) genotype data

To confirm relatedness in the pedigrees, reported relationships were compared with SNP array genotype data for all samples with available DNA (n = 31). All individuals were genotyped on the Infinium PsychArray BeadChip (PsychChip, Illumina, San Diego, CA, USA), in accordance with the manufacturer’s recommendations. Genotype data were analyzed using the integrated Illumina data analysis software platform GenomeStudiov2011.1. Quality control (QC) was performed using PLINK (http://zzz.bwh.harvard.edu/plink/download.shtml) [26], in accordance with the standard QC procedures of the Psychiatric Genomics Consortium (PGC) (https://sites.google.com/a/broadinstitute.org/psych-chip-resources/qc-methods#TOC-Standard-PGC-GWAS-QC). Imputation was conducted with IMPUTE2 [27, 28], using the 1000 Genomes Project phase 3 reference and standard parameters [29, 30].

### Polygenic risk scores (PRS)

PRS were calculated for all members of the two Cuban families with available SNP array genotype data. The calculations were performed using the summary statistics of the largest GWAS of BD to date. This GWAS was performed by the PGC, and comprised 20,352 cases and 31,358 controls [31]. After clumping of summary statistics based on best-guess genotype data, PRS were calculated using imputed dosage data in *R* v3.3 [32]. In all PRS computations, the allele with the positive effect direction was considered. The PRS therefore represent weighted, cumulative, additive risk. Cumulative PRS (sum of risk load) were calculated using 10 different *p*-value thresholds: <5×10^−8^, <1×10^−7^, <1×10^−6^, <1×10^‑5^, <1×10^−4^, <0.001, <0.01, <0.05, <0.1, and <0.2. PRS were scaled to represent relative risk load (minimum possible cumulative risk load = 0, maximum = 1). PRS analyses were conducted in *R* v3.3, using the function polygenic of the *R* package GenABEL, which implements a linear mixed model that takes family structure into account [33]. Gender was used as a covariate in the analysis. In a first step, residuals were calculated in the polygenic model using the formula *phenotype ~ covariates*, plus the genetic relationship matrix as a random effect. Residuals from this model were then used in a second linear model, with the formula *residuals ~ PRS*. In the second step, association test statistics with 95% confidence intervals (CI) were calculated using bootstrapping (*R* package boot, non-parametric bootstrapping using ordinary resampling with 2,000 replications) [34, 35]. In accordance with the hypothesis that family members with BD have increased PRS for BD compared to healthy relatives, one-sided *p*-values were calculated for all PRS-based analyses. Since the residuals were not normally distributed, *p*-values were confirmed using permutation analyses (10,000 permutations) (S2 Table).

### Exome sequencing and data analysis procedure

The exome sequencing step included a total of 15 BD cases (8 females, 7 males) from the two families. The three remaining BD cases were excluded. Two of these three individuals (individual 11, family 1; and individual 19, family 2) were excluded as they had been diagnosed with MDD in the initial 1997 assessment, and assigned a diagnosis of BD II in the reassessment in 2016, when exome sequencing in individuals with a BD diagnosis had already been performed. Individual 17 of family 2 was excluded on the grounds of being related by marriage only. The initial diagnoses of the BD cases, as well as the respective subtypes (BD I, II, or NOS), remained stable, and were confirmed during the second assessment. The SureSelect^XT^ Human All Exon V5 capture library from Agilent Technologies (Santa Clara, California, USA) was used for target enrichment. Exome sequencing was performed on an Illumina HiSeq2500 v4 system (San Diego, California, USA) using 2 x 125 bp paired-end sequencing. A brief technical report is provided in Table 1. The generated exome sequencing data are available at the European Genome-phenome Archive (EGA; https://ega-archive.org) under accession number EGAS00001003085.

**Table 1:** Exome sequencing: Technical report summary.

	Pedigree 1	Pedigrees 2, 3, and 4
**Number of covered genes**	21,522	
**Captured target regions (Mb)**	50	
**Mean coverage**	85.25x	92.90x
**Percentage of target regions with coverage** ≥ **30x**	92.55	91.33

Mb = Mega base pairs

For the analysis of pedigree data, the Varbank pipeline of the Cologne Center of Genomics was used (https://varbank.ccg.uni-koeln.de). This pipeline provides a user-friendly interface and incorporates a set of standard filters and annotations. The first filter was set to detect heterozygous variants with an allele read frequency of 25–75% relative to the reference. Variants with a minimum coverage of 10x and a quality score of 10 were included. The Varbank workflow includes the Genome Analysis Toolkit (GATK), and the GATK Best Practices workflow was applied. To decrease the number of specific pipeline artifacts, the default settings of the Varbank in-house database filter (comprising: epilepsy data, n = 511; and mixed case and control structural data, n = 611) were applied. The analysis of exome sequencing data focused on single-nucleotide variants (SNVs), as well as small insertions and deletions (InDels). The analysis was restricted to nonsynonymous variants with an effect on primary protein structure or a strong splice site effect. Focus was placed on variants that were shared by all BD cases within the respective pedigree.

To identify rare alleles, filtering for variants with a minor allele frequency (MAF) of < 0.1% was performed using the data of the Exome Aggregation Consortium (ExAC; http://exac.broadinstitute.org, version 0.3, 2015). For each variant, information concerning *in silico* predicted effects on protein structure was obtained from the database for nonsynonymous SNPs’ functional predictions (dbNSFP). This database provides functional prediction scores from various algorithms, and annotations of nonsynonymous SNVs [36, 37]. In accordance with Purcell et al., the analyses involved the commonly applied predictions of SIFT; Polyphen-2 (HumDiv- and HumVar-trained models); LRT; and MutationTaster [38]. Only variants that were predicted to have a potentially damaging (damaging/probably damaging/possibly damaging/deleterious/disease-causing) impact on protein structure by at least four of the five algorithms were included in subsequent analyses. Predictions for InDels were obtained from the tools MutationTaster and SIFT/Provean [39, 40]. InDels predicted to have a possible deleterious effect by at least one of the three tools were included in the subsequent analyses.

To verify the exome sequencing results, Sanger sequencing was performed using standard polymerase chain reaction conditions and a 3130xl Genetic Analyzer (Applied Biosystems, Foster City, California, USA). Primer sequences are available upon request.

To estimate possible population effects, Sanger sequencing of the identified variants was performed in all individuals with available DNA from the two unrelated Cuban families, including individuals related by marriage only. These sequencing data were also used to follow up the segregation of variants in each family.

### Brain-expression

To determine whether the identified candidate genes are expressed in the human brain, the Genotype-Tissue Expression (GTEx) Project portal (http://www.gtexportal.org/, on 05/11/2017) was accessed. Candidate genes with a Reads Per Kilobase per Million mapped reads (RPKM) of ≥ 0.1 in hippocampal tissue were classified as showing brain-expression.

### Non-parametric linkage analysis

The SNP array data were used to perform non-parametric linkage (NPL) analyses. NPL analysis is the method of choice for pedigree studies of complex genetic traits, since it is robust to uncertainty concerning the mode of inheritance [41]. NPL investigates whether affected individuals share alleles identity-by-descent more often than would be expected under a random segregation [41]. First, a QC of the SNP array data was performed using PLINK 1.9 (https://www.cog-genomics.org/plink2) [42]. SNPs were filtered for a call rate of at least 95% and an MAF of ≥ 5%. After linkage disequilibrium pruning with a window size of 500 kb and an r^2^ threshold of 0.01, a set of 9690 SNPs remained. These were used for subsequent NPL analyses of pedigree 1 and subpedigrees 2 and 3. In addition, we also performed NPL analyses for the 17 rare candidate variants identified in the exome sequencing step. The two NPL Z-score statistics NPL_all_ and NPL_pairs_ were computed using the MERLIN software version 1.1.2 [43, 44]. Both the standard linear model and the exponential likelihood model [45] implemented in MERLIN were applied to generate model-free LOD scores. Here, a strict phenotype definition was applied, whereby subjects with a diagnosis of BD I, BD II or BD NOS were classified as “affected”; healthy individuals were classified as “unaffected” and individuals with other psychiatric phenotypes were classified as “unknown”.

### Population substructure analysis

For the population substructure analysis, post-QC pre-imputation SNP array data were used. The analysis was carried out using PLINK 1.9 [42] and *R*. First, genetic data from the 1000 Genomes Project Phase 3 reference panel [29, 30] were converted to the PLINK binary format and merged with the genotype data of the Cuban pedigrees. Second, the combined dataset was subjected to the following three variant filtering steps: (i) removal of variants with an MAF of < 0.05 or a HWE *p*-value of < 10^-3^; (ii) removal of variants mapping to the extended MHC region (chromosome 6, 25–35 Mb) or to a typical inversion site on chromosome 8 (7–13 Mb); and (iii) LD pruning (command—*indep-pairwise 200 100 0*.*2*). Next, the pairwise identity-by-state matrix of all individuals was calculated using the command—*genome* on the filtered genotype data. A multidimensional scaling (MDS) analysis was then performed on the identity-by-state matrix using the eigendecomposition-based algorithm in PLINK.

Populations from 1000 Genomes were selected for the analysis using backward elimination, starting with all populations and retaining only the ones to which Cuban family members showed close clustering. The final population selection was: African ancestry in Barbados and the USA (ACB, ASW); Puerto Rican (PUR); Spanish (IBS); and Tuscan Italian (TSI). In an MDS analysis, high relatedness between family members leads to artifacts. To avoid such artifacts, only one representative subject of each highly related cluster was included in the analysis. Therefore, data on only 19 of the 31 family members are displayed (S1 Fig).

## Results

### Relatedness and polygenic risk scores based on SNP genotype data

SNP array genotyping data confirmed the pedigree relationships depicted in Fig 1.

After correction for multiple testing, PRS based on BD-associated genetic variants were not significantly higher in BD cases than in family members with no lifetime history of psychiatric illness (S2 Fig). When PRS calculation was restricted to BD variants with genome-wide significance (*p*-value threshold 5×10^−8^), the PRS of BD cases from the two Cuban families showed a nominally significant increase compared to the PRS of family members with no psychiatric disorder or a non-BD psychiatric phenotype (*p* = 0.027, S2B Fig). Since affected family members showed no significant increase in the burden of common variants, an analysis of rare variants in these patients was considered a promising approach.

### Pedigree information and technical summary

To identify rare variants with a potential contribution to BD susceptibility, a total of 15 individuals with BD I, BD II, or BD NOS were selected from the two Cuban families for whole exome sequencing. These comprised four subjects from family 1 (Fig 1A); and 11 subjects from family 2 (Fig 1B). Mean coverage ranged from 75x to 116x. On average, 91.5% of the target regions had a minimum coverage of 30x (Table 1). Family 2 was divided into subpedigrees 2, 3, and 4. Filtering and rare variant identification was performed separately for each subpedigree.

### Analysis of exome sequencing data and rare variant identification

Application of the Varbank pipeline of the Cologne Center for Genomics (https://varbank.ccg.uni-koeln.de) led to the identification of an average of 1,019 variants with an MAF of < 1% per exome. A total of 20, 30, and 126 variants were shared by BD cases in pedigrees 1, 2, and 3, respectively. To identify variants of potentially higher penetrance, the analysis was restricted to very rare variants with an MAF of < 0.1%. This reduced the number of shared variants in the aforementioned pedigrees to 9, 14, and 59, respectively. Pedigrees 2 and 3 are unrelated, and gave rise to bilineal pedigree 4. Thus, none of the variants were shared by all BD cases from pedigree 4.

To prioritize the identified variants, filtering was performed for alleles that were potentially functional and predicted to be potentially damaging by at least four of five (SNVs) or one of three (InDels) of the applied prediction tools. This reduced the number of rare variants to 2, 3, and 12 variants in the respective pedigrees. These 17 candidate variants comprised 14 missense variants; and 3 small InDels, which all lead to a frameshift (Table 2).

**Table 2:** Rare exome variants identified in affected individuals from the investigated Cuban pedigrees.

Pedigree 1								Predicted effects on protein function				
Gene	Chr	Position	Ref	Alt	ExAC MAF	dbSNP141	AA change	SIFT	PPh-2 HumDIV	PPh-2 HumVAR	LRT	Mutation Taster
*SERPING1*	11	57379205	C	T	4.9E-05	rs141075266	p.L349F	**D**	**P**	B	**D**	**D**
*TMEM220*	17	10633149	C	A	NA	NA	p.R7L	**D**	**D**	**P**	N	**D**
**Pedigree 2**												
*ABCA4*	1	94544977	A	T	4.9E-04	rs61748549	p.N380K	**D**	**P**	**P**	**D**	**D**
*DNAH7*	2	196726484	C	T	4.2E-04	rs201185180	p.E2565K	**D**	**D**	**P**	**D**	**D**
*RCCD1*	15	91500673	G	C	NA	NA	p.G166A	**D**	**D**	**P**	N	**D**
**Pedigree 3**												
*EPS8L3*	1	110293386	C	T	3.1E-04	rs148185176	p.G526R	**D**	**D**	**D**	**D**	**D**
*OLFML2B*	1	161953665	C	A	3.0E-04	rs142349285	p.A685S	**D**	**D**	**D**	NA	**D**
*CAPN2*	1	223934845	C	T	6.0E-04	rs140704789	p.S236F	**D**	**D**	**D**	**D**	**D**
*COL3A1*	2	189875383	G	A	2.0E-04	rs140646380	p.G1341S	T	**D**	**D**	**D**	**D**
*ATR*	3	142281560	T	TT	NA	NA	p.L229Tfs*13	NA	NA	NA	NA	**D**
*CSNK1G3*	5	122926124	C	T	NA	NA	p.R288C	**D**	**P**	**P**	**D**	**D**
*THYN1*	11	134119132	G	A	9.9E-05	rs143669769	p.H137Y	T	**P**	**P**	**D**	**D**
*MYH7*	14	23898481	-	TG	NA	NA	p.K405Nfs*17	NA	NA	NA	NA	**D**
*FAM169B*	15	98995065	A	G	4.6E-04	rs183490372	p.M120T	**D**	**D**	**D**	**D**	**D**
*ZNF433*	19	12127214	AGAGG	-	NA	NA	p.S155Cfs*5	NA	NA	NA	NA	**D**
*CRX*	19	48337728	C	G	4.1E-04	rs139340178	p.H10D	T	**D**	**P**	**D**	**D**
*SELENOO*	22	50648648	G	T	2.5E-05	NA	p.Q326H	**D**	**D**	**D**	**D**	**D**

A broader variant list is provided in the S3 Table. This was generated using relaxed filter criteria (MAF ≤ 5% according to ExAC and predicted to be potentially damaging by at least one of the five prediction tools).

Variants identified in pedigree 1 and subpedigrees 2 and 3. Bold formatting indicates alleles with a predicted potentially damaging effect on protein structure or function. Variants are sorted according to chromosomal position. Abbreviations: Chr, chromosome; Position, chromosomal position of respective variant according to hg19/GRCh37; Ref, reference allele; Alt, alternative allele; ExAC MAF, minor allele frequency according to the data of the Exome Aggregation Consortium; AA change, amino acid change; PPh-2, PolyPhen-2; D, damaging/probably damaging/deleterious/disease-causing; P, possibly damaging; B, benign; T, tolerated; N, neutral; NA, not available.

### Segregation analysis

In the segregation analysis, testing for all 17 identified rare variants with potentially damaging effects on protein function was performed in all family members with available DNA from both families (Tables 3–5).

**Table 3:** Results of the segregation analysis of sequence variants in pedigree 1 as well as brain expression of the implicated genes and data from the NPL analyses.

Pedigree 1						NPL	
Gene	Codon change	BD *n* = *5*	Other psychiatric disorders *n* = 1	Unaffected *n = 3*	Expression RPKM	exLOD	*p-*value
***TMEM220***	C > A	5	1	2	**0.98**	1.08	0.01
***SERPING1***	C > T	4	0	1	**8.11**	0.02	0.37

Information on genotypes at variant sites for relatives in pedigree 1. Individuals related by marriage only were excluded.

*N*, total number of individuals within each phenotype group. Numbers indicate how often the variants were found in the tested family members, as categorized according to phenotype. BD, Bipolar disorder types: BD I; BD II; and BD not otherwise specified. Other psychiatric disorders: recurrent major depressive disorder (MDD); MDD single episode; and alcohol abuse. Unaffected: healthy individuals; NPL, non-parametric linkage using the NPL_all_ Z-score statistics; exLOD, logarithm of the odds (exponential model); *p*-value, probability of exLOD. Bold formatting indicates Reads Per Kilobase Million (RPKM) ≥ 0.1.

**Table 4:** Results of the segregation analysis of sequence variants in subpedigrees 2 and 4 as well as brain expression of the implicated genes and data from the NPL analyses.

		Pedigree 2	Pedigree 4		NPL (Pedigree 2)	
Gene	Codon change	BD *n* = 4	BD *n* = 5	Expression RPKM	exLOD	*p-*value
***RCCD1***	G > C	4	2	**3.17**	-0.26	0.86
***DNAH7***	C > T	4	2	**0.42**	-0.11	0.76
*ABCA4*	A > T	4	2	0.06	-0.18	0.82

Information on genotypes at variant sites for the individuals in subpedigree 2 and the bilineal offspring in subpedigree 4. Individuals related by marriage only were excluded.

*N*, total number of individuals within each phenotype group. Numbers indicate how often the variants were found in the tested family members, as categorized according to phenotype. BD, Bipolar disorder types: BD I; BD II; and BD not otherwise specified. NPL, non-parametric linkage using the NPL_all_ Z-score statistics; exLOD, logarithm of the odds (exponential model); *p*-value, probability of exLOD. Bold formatting indicates Reads Per Kilobase Million (RPKM) ≥ 0.1.

**Table 5:** Results of the segregation analysis of sequence variants in subpedigrees 3 and 4 as well as brain expression of the implicated genes and data from the NPL analyses.

		Pedigree 3	Pedigree 4	Pedigrees 3+4			NPL (Pedigree 3)	
Gene	Codon change	BD *n* = 3	BD *n* = 5	Other psychiatric disorders *n* = 2	Unaffected *n* = 3	Expression RPKM	exLOD	*p*-value
***COL3A1***	G > A	3	4	1	2	**0.52**	0.60	0.05
***THYN1***	G > A	3	3	1	2	**20.93**	0.30	0.12
*EPS8L3*	G > C	3	3	2	3	0.01	0.60	0.05
***CAPN2***	C > T	3	2	1	1	**9.26**	0.09	0.26
***ATR***	T > TT	3	2	1	2	**1.49**	0.60	0.05
***MYH7***	- > TG	3	2	2	1	**0.92**	0.09	0.26
***ZNF433***	AGAGG > -	3	2	0	2	**0.82**	0.09	0.26
***OLFML2B***	C > A	3	2	0	2	**0.71**	0.09	0.26
*FAM169B*	A > G	3	2	1	1	0.01	0.09	0.26
***CSNK1G3***	C > T	3	1	1	1	**3.56**	0.09	0.26
*CRX*	C > G	3	1	2	2	0	0.09	0.26
***SELENOO***	G > T	3	0	2	1	**8.47**	0.09	0.26

Information on genotypes at variant sites for the individuals of subpedigree 3 and the bilineal offspring in subpedigree 4. Individuals related by marriage only were excluded. Variants were ranked in descending order of occurrence in pedigree 4.

*N*, total number of individuals within each phenotype group. Numbers indicate how often the variants were found in the tested family members, as categorized according to phenotype. BD, Bipolar disorder types: BD I; BD II; and BD not otherwise specified. Other psychiatric disorders: recurrent major depressive disorder (MDD); MDD single episode; and alcohol abuse. Unaffected: healthy individuals; NPL, non-parametric linkage using the NPL_all_ Z-score statistics; exLOD, logarithm of the odds (exponential model); *p*-value, probability of exLOD. Bold formatting indicates Reads Per Kilobase Million (RPKM) ≥ 0.1.

In pedigree 1 (Table 3), one rare variant located in the gene *TMEM220* segregated with all five BD cases across four generations (Fig 1A; individuals 4, 5, 7, 9, and 11). This variant was also identified in two healthy relatives (Fig 1A; individuals 1 and 3), and one relative with alcohol abuse who had two children with a history of MDD (Fig 1A, individual 10).

The second candidate variant (rs141075266) was located in the *SERPING1* gene. This variant segregated with BD in the four BD cases who had undergone exome sequencing, but was not present in the BD-affected offspring in the fifth generation (Fig 1A, individual 11). The variant was also identified in a healthy parent in the third generation, who was an obligate carrier (Fig 1A, individual 3).

In pedigree 2 (Table 4), three rare variants were identified in all BD cases: *RCCD1*; *DNAH7* (rs201185180); and *ABCA4* (rs61748549). Further evaluation of segregation in pedigree 2 was precluded by the absence of additional individuals with available DNA.

In pedigree 3 (Table 5), none of the candidate variants identified in the exome-sequenced BD cases showed complete penetrance, since they were also detected in at least one of three healthy relatives (Fig 1B; individuals 7, 8, and 20). Interestingly, the variants identified in *EPS8L3* (rs148185176), *MYH7*, *CRX* (rs139340178), and *SELENOO*, were also present in two relatives with alcohol abuse (Fig 1B, individuals 6 and 18).

For candidate variants identified in pedigrees 2 and 3, segregation was also investigated in four affected siblings from bilineal pedigree 4. In three of these four siblings, two missense variants in the genes *COL3A1* and *THYN1* were detected (Fig 1B; individuals 11, 12, 14, and 15). Individual 14 had transmitted the *COL3A1* variant to their BD II-affected offspring (Fig 1B; individual 19). On average, the remaining variants were present in 50% of the siblings and their offspring individual 19, in accordance with Mendelian expectation. One exception was the variant located in the gene *SELENOO*, which was not transmitted to any of the siblings. Tables 3–5 provide both a summary of these analyses, and details of the prioritized variants in the two multigenerational Cuban families.

All candidate variants identified in one pedigree were then sequenced in the other pedigrees and in relatives-by-marriage. None of the variants were found in the other family, or in subjects related by marriage only.

### Brain-expression

The prioritized rare variants were located in 17 genes. According to the GTEx Project portal, a total of 13 of these genes (*SERPING1*, *TMEM220*, *MYH7*, *THYN1*, *ATR*, *CSNK1G3*, *ZNF433*, *OLFML2B*, *CAPN2*, *RCCD1*, *DNAH7*, *SELENOO*, *and COL3A1)* show brain-expression (Tables 3–5, brain-expressed genes shown in bold).

### Non-parametric linkage analysis

Linkage analysis did not reveal any suggestive linkage signal exceeding the threshold of LOD > 2.2 [22] in any of the four applied approaches (NPL_pairs_ and NPL_all_, linear and exponential likelihood model). This was attributable to the fact that the maximum achievable LOD scores were 1.47 in pedigree 1, 1.75 in subpedigree 2, and 0.60 in subpedigree 3 (NPL_all_, exponential likelihood model). For each rare, segregating candidate variant identified in the exome sequencing step, we calculated the LOD score of the NPL_all_ exponential likelihood model (exLOD) and the corresponding *p*-value (Tables 3–5). In addition, exLOD scores and corresponding *p*-values of the markers closest to the variants with an MAF of ≤ 5% and predicted to be potentially damaging by at least one of the five prediction tools are shown in S3 Table.

### Population substructure analysis

Pedigree 1 mainly clustered with admixed Americans (PUR). However, two individuals also showed a degree of African admixture (ACB and ASW). Subpedigrees 2, 3, and 4 clustered with both Europeans (TSI and IBS) and admixed Americans. Married-in individuals mainly clustered with admixed Americans, with two married-in subjects showing differing degrees of African admixture (S1 Fig).

## Discussion

In the present cohort, the PRS of BD cases, representing the genetic risk load for common BD-associated variants, were not significantly higher than the PRS of their unaffected relatives. This may suggest that BD susceptibility in the investigated Cuban pedigrees may have been conferred by highly penetrant genetic factors, rather than through polygenic effects. However, the results do not exclude the possibility that the high prevalence of BD in these families was attributable to the collective contribution of common variants, since the present approach had three limitations. First, no optimal *p*-value threshold has yet been established for the calculation of PRS on the basis of the—as yet unpublished—PGC GWAS of BD. The present analyses therefore applied ten different *p*-value thresholds for the inclusion of variants, and the significance threshold was adapted for ten tests. PRS calculated on the basis of variants with genome-wide significance showed a nominally significant increase in BD cases. However, this result did not withstand correction for multiple testing. Second, the sample size for the PRS analysis was small (17 BD cases and 6 unaffected individuals), which resulted in limited statistical power. Third, if a polygenic contribution is present in families, unaffected family members may also show an increased PRS, while not having developed the disease due to the absence of other risk factors. To investigate this hypothesis, a comparison between unaffected family members from the present cohort and unrelated controls from the southeastern Cuban population is warranted.

The present whole exome sequencing approach identified 17 nonsynonymous, potentially functional, and rare variants with a potential contribution to BD development. All of these rare alleles showed incomplete penetrance for BD. This finding is unsurprising, since even large copy number variants—which represent the strongest known genetic risk factors for psychiatric disorders at the time of writing—show reduced penetrance [46].

The most promising finding of the present study was the rare coding variant in *SERPING1*, which was identified in pedigree 1. This gene is brain expressed and encodes the serpin peptidase inhibitor clade G member 1, a highly glycosylated plasma protein implicated in the complement cascade. The protein inhibits C1r and C1s of the first complement component, and thus regulates complement activation [47]. BD has shown a substantial genetic correlation of around 68% with schizophrenia and, interestingly, *SERPING1* was among the genome-wide significant risk loci for schizophrenia identified in the largest GWAS of schizophrenia to date (rs9420, *p* = 2.24×10^−9^) [12, 48]. Furthermore, the complement cascade has recently been identified as an important pathway through which rare CNVs could modify risk for schizophrenia by influencing the efficiency of synaptic pruning [49]. The variant identified in the present study leads to a leucine to phenylalanine (p.L349F) substitution within a beta strand (http://www.uniprot.org), and was predicted to have a deleterious effect on protein function by four of the five applied algorithms. *SERPING1* is also a risk gene for hereditary angioedema (HAE). However, the present variant is not listed as a causal HAE mutation in established clinical databases [50]. In addition, no clinical features of HAE were reported in pedigree 1. Interestingly, a recent study has suggested a possible link between HAE and depression [51]. This association might be attributable to the chronic and life-threatening nature of HAE. Alternatively, it might suggest a potential pleiotropic effect secondary to the impact of rare variants.

The second variant in pedigree 1 was identified in *TMEM220*. At the time of writing, this gene is poorly characterized, and has no known role in any neuropsychiatric disorder.

In family 2, rare, potentially functional variants implicated a total of 15 candidate genes. Of these, 11 show brain-expression.

No overlap was found between the 17 potential candidate genes in the present study and findings from recent next generation sequencing studies of BD [15, 17, 19, 20, 22–24, 52]. This may reflect the pronounced genetic heterogeneity of BD; the variety of implicated pathways; ethnic differences as well as methodological differences between the respective studies, which applied differing variant prioritization approaches.

To generate further evidence for the role of the identified candidate variants in BD etiology, comprehensive analyses of data from further family- and replication studies are necessary. This could be achieved by combining exome sequencing data from international consortia, such as the Bipolar Sequencing Consortium [53]. In addition, the investigation of families with distinct subphenotypes may reduce genetic heterogeneity, and generate further insights into the observed differences in clinical presentation [20, 54].

In the present analyses, data from the ExAC (http://exac.broadinstitute.org, version 0.3, 2015) were used to determine the MAF of all identified variants. ExAC compiles and harmonizes exome sequencing data from a variety of projects and cohorts, and comprises the exomes of 60,706 unrelated individuals. ExAC represents the largest collection of human exome data to date. The majority of ExAC data originate from Europe (60%). A lower proportion of the data originates from Africa (9%) and Latin America (10%). A recent admixture analysis demonstrated that the genetic ancestry of the Cuban population is 72% European, 20% African, and 8% Native American [55]. This is in line with the results of the present population substructure analysis, which indicated that the investigated pedigrees mainly clustered with Southern European and admixed American ethnicities. The ExAC data were therefore considered appropriate in terms of estimating the MAF of variants identified in the present cohort. However, future studies are warranted to determine common and rare variant profiles in the Cuban population, a step which was beyond the scope of the present study.

All candidate variants identified in one pedigree were then tested in the other pedigrees and in individuals related by marriage only. According to the present sequencing data, none of the variants were found in both families or in subjects related by marriage. This provides further support for the hypothesis that the identified variants are indeed rare in the Cuban population. However, in view of the small sample size, the possibility that the deviations in MAF may have been attributable to population stratification cannot be excluded.

Candidate gene selection in the present study was based on a filtering and prioritization procedure. Although the applied prediction algorithms are widely used, their assessment of the potential impact of a given variant on protein structure and function has certain limitations. The applied approaches attempt to prioritize variants on the basis of their population allele frequencies and measures of conservation at the phylogenetic level, and/or in terms of amino acid properties [40, 56–58]. However, functional studies of the identified rare variants are required before more definite conclusions on their functional consequences can be drawn.

The focus of the present study was the identification of rare variants (MAF < 0.1%) with high penetrance. Since rare and low-frequency variants with higher MAFs (0.1–5%) and moderate effects may also contribute to BD development, a second filtering step was performed using relaxed criteria. The broader variant list generated in this step is presented in S3 Table including linkage evidence from the respective regions. To assess their contribution to BD etiology, these variants require evaluation in future studies.

In conclusion, rare, potentially functional variants implicated a total of 17 genes in two multiply affected BD families from Cuba. These genes may thus play a role in BD etiology. The most promising variant was located in the gene *SERPING1*, which was reported as a genome-wide significant risk gene for schizophrenia in previous research. The present data therefore suggest novel candidate genes for BD susceptibility, and may facilitate the discovery of disease-relevant pathways and regulatory networks.

## Supporting information

S1 Table Additional clinical information for individuals selected for whole exome sequencing. Overview of bipolar disorder (BD) subtype and psychosis-status of the investigated individuals. Age-at-onset is reported in 5-year intervals.
(XLSX)

S2 Table Analysis of association between polygenic risk scores (PRS) and bipolar disorder (BD). Association test statistics including percentile-based 95% bootstrap confidence intervals (CI) for each of the ten *p*-value thresholds. Because of deviations from normality, one-sided *p*-values were confirmed using permutation-based analyses (10,000 permutations). *R*^*2*^ = coefficient of determination, SE = standard error.
(XLSX)

S3 Table Overview of variants with an MAF ≤ 5% that were predicted to be potentially damaging by at least one of the five tools. Genetic variants with an MAF of ≤ 5% that were predicted to be potentially damaging by at least one of the five applied algorithms are shown in S3 Table. The identified variants were shared by all investigated BD cases in the respective pedigree.
(XLSX)

S1 Fig Population substructure analysis. Multidimensional scaling (MDS) components 1 and 2 are displayed. The axes have been scaled to show standard deviations. Red diamonds indicate individuals from pedigree 1. Blue diamonds indicate individuals from subpedigrees 2, 3, and 4. Green diamonds indicate individuals related by marriage only. Diamond size is scaled to indicate the number of subjects represented (n = 1–9 per diamond). 1000 Genomes Project population codes: ACB, African Caribbeans in Barbados (pink); ASW, Americans of African Ancestry in Southwest USA (purple); PUR, Puerto Ricans from Puerto Rico (yellow); TSI, Toscani in Italia (brown); IBS, Iberian Population in Spain (orange).
(TIF)

S2 Fig Visual depiction of association results from S2 Table. (A) Graphical depiction of test statistics for the association of BD PRS with BD diagnosis, as shown in S2 Table. Association strength is illustrated via *p*-values on a negative-logarithmic scale including percentile-based 95% bootstrap confidence intervals (CI) for each of the ten PRS based on different *p*-value thresholds. Nominal significance is indicated by an orange line (-log10(*p*) = 1.3), and the significance threshold after Bonferroni-correction for ten tests is indicated by a green line (-log10(*p*) = 2.3). The respective coefficient of determination R^2^ is shown at the top of the plot. (B) Boxplots of scaled BD PRS for two *p*-value thresholds, 5×10^−8^ and 0.05. White box represents healthy individuals, orange box represents BD cases, and blue box represents other psychiatric phenotypes.
(TIF)

S1 Text Members of the Bipolar Disorder Working Group of the Psychiatric Genomics Consortium. (DOCX)

## References

[1] Nöthen MM, Nieratschker V, Cichon S, Rietschel M. New findings in the genetics of major psychoses. Dialogues Clin Neurosci. 2010;12(1):85–93. 20373670 10.31887/DCNS.2010.12.1/mnoethen PMC3181946

[2] Craddock N, Jones I. Genetics of bipolar disorder. J Med Genet. 1999;36(8):585–94. 10465107 10.1136/jmg.36.8.585 PMC1762980

[3] Mühleisen TW, Leber M, Schulze TG, Strohmaier J, Degenhardt F, Treutlein J, et al Genome-wide association study reveals two new risk loci for bipolar disorder. Nat Commun. 2014;5 10.1038/ncomms4339 24618891

[4] Whiteford HA, Ferrari AJ, Degenhardt L, Feigin V, Vos T. The Global Burden of Mental, Neurological and Substance Use Disorders: An Analysis from the Global Burden of Disease Study 2010. Plos One. 2015;10(2). doi: ARTN e0116820 10.1371/journal.pone.0116820 25658103 PMC4320057

[5] Lichtenstein P, Yip BH, Björk C, Pawitan Y, Cannon TD, Sullivan PF, et al Common genetic determinants of schizophrenia and bipolar disorder in Swedish families: a population-based study. The Lancet. 2009;373(9659):234–9. 10.1016/S0140-6736(09)60072-6 19150704 PMC3879718

[6] Baum AE, Akula N, Cabanero M, Cardona I, Corona W, Klemens B, et al A genome-wide association study implicates diacylglycerol kinase eta (DGKH) and several other genes in the etiology of bipolar disorder. Mol Psychiatry. 2008;13(2):197–207. 10.1038/sj.mp.4002012 17486107 PMC2527618

[7] Cichon S, Mühleisen TW, Degenhardt FA, Mattheisen M, Miro X, Strohmaier J, et al Genome-wide Association Study Identifies Genetic Variation in Neurocan as a Susceptibility Factor for Bipolar Disorder. Am J Hum Genet. 2011;88(3):372–81. 10.1016/j.ajhg.2011.01.017 21353194 PMC3059436

[8] Ferreira MAR, O'Donovan MC, Meng YA, Jones IR, Ruderfer DM, Jones L, et al Collaborative genome-wide association analysis supports a role for ANK3 and CACNA1C in bipolar disorder. Nat Genet. 2008;40(9):1056–8. 10.1038/ng.209 18711365 PMC2703780

[9] Chen DT, Jiang X, Akula N, Shugart YY, Wendland JR, Steele CJM, et al Genome-wide association study meta-analysis of European and Asian-ancestry samples identifies three novel loci associated with bipolar disorder. Mol Psychiatry. 2013;18(2):195–205. 10.1038/mp.2011.157 22182935

[10] Psychiatric GWAS Consortium Bipolar Disorder Working Group. Large-scale genome-wide association analysis of bipolar disorder identifies a new susceptibility locus near ODZ4. Nat Genet. 2011;43(10):977–83. 10.1038/ng.943 PMCID: PMC3637176. 21926972 PMC3637176

[11] Nurnberger JI Jr., Koller DL, Jung J, Edenberg HJ, Foroud T, Guella I, et al Identification of pathways for bipolar disorder: a meta-analysis. JAMA Psychiatry. 2014;71(6):657–64. 10.1001/jamapsychiatry.2014.176 PMCID: PMC4523227. 24718920 PMC4523227

[12] Cross-Disorder Group of the Psychiatric Genomics Consortium. Genetic relationship between five psychiatric disorders estimated from genome-wide SNPs. Nature Genetics. 2013;45(9):984–94. 10.1038/ng.2711 23933821 PMC3800159

[13] Lee Sang H, Wray Naomi R, Goddard Michael E, Visscher Peter M. Estimating Missing Heritability for Disease from Genome-wide Association Studies. Am J Hum Genet. 2011;88(3):294–305. 10.1016/j.ajhg.2011.02.002 21376301 PMC3059431

[14] Collins AL, Kim Y, Szatkiewicz JP, Bloom RJ, Hilliard CE, Quackenbush CR, et al Identifying Bipolar Disorder Susceptibility Loci in a Densely Affected Pedigree. Mol Psychiatry. 2013;18(12):1245–6. 10.1038/mp.2012.176 23247078 PMC3899577

[15] Kataoka M, Matoba N, Sawada T, Kazuno AA, Ishiwata M, Fujii K, et al Exome sequencing for bipolar disorder points to roles of de novo loss-of-function and protein-altering mutations. Mol Psychiatry. 2016;21(7):885–93. 10.1038/mp.2016.69 27217147 PMC5414074

[16] Kato T. Whole genome/exome sequencing in mood and psychotic disorders. Psychiatry Clin Neurosci. 2015;69(2):65–76. 10.1111/pcn.12247 25319632

[17] Goes FS, Pirooznia M, Parla JS, Kramer M, Ghiban E, Mavruk S, et al Exome Sequencing of Familial Bipolar Disorder. JAMA Psychiatry. 2016;73(6):590–7. 10.1001/jamapsychiatry.2016.0251 PMCID: PMC5600716. 27120077 PMC5600716

[18] Ament SA, Szelinger S, Glusman G, Ashworth J, Hou L, Akula N, et al Rare variants in neuronal excitability genes influence risk for bipolar disorder. PNAS. 2015;112(11):3576–81. 10.1073/pnas.1424958112 25730879 PMC4371952

[19] Chen Y-C, Carter H, Parla J, Kramer M, Goes FS, Pirooznia M, et al A Hybrid Likelihood Model for Sequence-Based Disease Association Studies. PLoS Genet. 2013;9(1). 10.1371/journal.pgen.1003224 23358228 PMC3554549

[20] Cruceanu C, Ambalavanan A, Spiegelman D, Gauthier J, Lafrenière RG, Dion PA, et al Family-based exome-sequencing approach identifies rare susceptibility variants for lithium-responsive bipolar disorder1. Genome. 2013;56(10):634–40. 10.1139/gen-2013-0081 24237345

[21] Fiorentino A, O'Brien NL, Locke DP, McQuillin A, Jarram A, Anjorin A, et al Analysis of ANK3 and CACNA1C variants identified in bipolar disorder whole genome sequence data. Bipolar Disord. 2014;16(6):583–91. 10.1111/bdi.12203 PMCID: PMC4227602. 24716743 PMC4227602

[22] Georgi B, Craig D, Kember RL, Liu W, Lindquist I, Nasser S, et al Genomic view of bipolar disorder revealed by whole genome sequencing in a genetic isolate. PLoS Genet. 2014;10(3):e1004229 10.1371/journal.pgen.1004229 PMCID: PMC3953017. 24625924 PMC3953017

[23] Kerner B, Rao AR, Christensen B, Dandekar S, Yourshaw M, Nelson SF. Rare Genomic Variants Link Bipolar Disorder with Anxiety Disorders to CREB-Regulated Intracellular Signaling Pathways. Front Psychiatry. 2013;4 10.3389/fpsyt.2013.00154 24348429 PMC3842585

[24] Strauss KA, Markx S, Georgi B, Paul SM, Jinks RN, Hoshi T, et al A population-based study of KCNH7 p.Arg394His and bipolar spectrum disorder. Hum Mol Genet. 2014 10.1093/hmg/ddu335 24986916 PMC4222358

[25] Miller SA, Dykes DD, Polesky HF. A simple salting out procedure for extracting DNA from human nucleated cells. Nucl Acids Res. 1988;16(3):1215 3344216 10.1093/nar/16.3.1215 PMC334765

[26] Purcell S, Neale B, Todd-Brown K, Thomas L, Ferreira MA, Bender D, et al PLINK: a tool set for whole-genome association and population-based linkage analyses. Am J Hum Genet. 2007;81(3):559–75. 10.1086/519795 PMCID: 1950838. 17701901 PMC1950838

[27] Howie B, Marchini J, Stephens M. Genotype imputation with thousands of genomes. G3 (Bethesda). 2011;1(6):457–70. 10.1534/g3.111.001198 PMCID: 3276165. 22384356 PMC3276165

[28] Howie BN, Donnelly P, Marchini J. A flexible and accurate genotype imputation method for the next generation of genome-wide association studies. PLoS Genet. 2009;5(6):e1000529 10.1371/journal.pgen.1000529 PMCID: 2689936. 19543373 PMC2689936

[29] Sudmant PH, Rausch T, Gardner EJ, Handsaker RE, Abyzov A, Huddleston J, et al An integrated map of structural variation in 2,504 human genomes. Nature. 2015;526(7571):75–81. 10.1038/nature15394 PMCID: 4617611. 26432246 PMC4617611

[30] The 1000 Genomes Project Consortium, Auton A, Brooks LD, Durbin RM, Garrison EP, Kang HM, et al A global reference for human genetic variation. Nature. 2015;526(7571):68–74. 10.1038/nature15393 PMCID: 4750478. 26432245 PMC4750478

[31] Stahl E, Forstner A, McQuillin A, Ripke S, Ophoff R, Scott L, et al Genomewide association study identifies 30 loci associated with bipolar disorder. bioRxiv. 2017 10.1101/173062

[32] R Development Core Team. R: A Language and Environment for Statistical Computing. 2017.

[33] Belonogova NM, Svishcheva GR, van Duijn CM, Aulchenko YS, Axenovich TI. Region-based association analysis of human quantitative traits in related individuals. PLoS One. 2013;8(6):e65395 10.1371/journal.pone.0065395 PMCID: 3684601. 23799013 PMC3684601

[34] Canty A, Ripley BD. boot: Bootstrap R (S-Plus) Functions. 2017.

[35] Davison AC, Hinkley DV. Bootstrap Methods and Their Application. Cambridge: Cambridge University Press; 1997.

[36] Liu X, Jian X, Boerwinkle E. dbNSFP v2.0: A Database of Human Non-synonymous SNVs and Their Functional Predictions and Annotations. Human Mutation. 2013;34(9):E2393–E402. 10.1002/humu.22376 23843252 PMC4109890

[37] Liu X, Jian X, Boerwinkle E. dbNSFP: A lightweight database of human nonsynonymous SNPs and their functional predictions. Human Mutation. 2011;32(8):894–9. 10.1002/humu.21517 21520341 PMC3145015

[38] Purcell SM, Moran JL, Fromer M, Ruderfer D, Solovieff N, Roussos P, et al A polygenic burden of rare disruptive mutations in schizophrenia. Nature. 2014;506(7487):185–90. 10.1038/nature12975 24463508 PMC4136494

[39] Choi Y, Sims GE, Murphy S, Miller JR, Chan AP. Predicting the Functional Effect of Amino Acid Substitutions and Indels. PLoS ONE. 2012;7(10):e46688 10.1371/journal.pone.0046688 23056405 PMC3466303

[40] Schwarz JM, Cooper DN, Schuelke M, Seelow D. MutationTaster2: mutation prediction for the deep-sequencing age. Nat Meth. 2014;11(4):361–2. 10.1038/nmeth.2890 24681721

[41] Kruglyak L, Daly MJ, Reeve-Daly MP, Lander ES. Parametric and nonparametric linkage analysis: a unified multipoint approach. Am J Hum Genet. 1996;58(6):1347–63. PMCID: PMC1915045. 8651312 PMC1915045

[42] Chang CC, Chow CC, Tellier LC, Vattikuti S, Purcell SM, Lee JJ. Second-generation PLINK: rising to the challenge of larger and richer datasets. Gigascience. 2015;4:7 10.1186/s13742-015-0047-8 PMCID: PMC4342193. 25722852 PMC4342193

[43] Abecasis GR, Cherny SS, Cookson WO, Cardon LR. Merlin—rapid analysis of dense genetic maps using sparse gene flow trees. Nat Genet. 2002;30(1):97–101. 10.1038/ng786 11731797

[44] Whittemore AS, Halpern J. A class of tests for linkage using affected pedigree members. Biometrics. 1994;50(1):118–27. 8086596

[45] Kong A, Cox NJ. Allele-sharing models: LOD scores and accurate linkage tests. Am J Hum Genet. 1997;61(5):1179–88. 10.1086/301592 PMCID: PMC1716027. 9345087 PMC1716027

[46] CNV and Schizophrenia Working Groups of the Psychiatric Genomics Consortium. Contribution of copy number variants to schizophrenia from a genome-wide study of 41,321 subjects. Nat Genet. 2017;49(1):27–35. 10.1038/ng.3725 PMCID: PMC5737772. 27869829 PMC5737772

[47] Johnsrud I, Kulseth MA, Rødningen OK, Landrø L, Helsing P, Waage Nielsen E, et al A Nationwide Study of Norwegian Patients with Hereditary Angioedema with C1 Inhibitor Deficiency Identified Six Novel Mutations in SERPING1. PLoS ONE. 2015;10(7). 10.1371/journal.pone.0131637 26154504 PMC4496036

[48] Schizophrenia Working Group of the Psychiatric Genomics Consortium. Biological insights from 108 schizophrenia-associated genetic loci. Nature. 2014;511(7510):421–7. 10.1038/nature13595 PMCID: 4112379. 25056061 PMC4112379

[49] Sekar A, Bialas AR, de Rivera H, Davis A, Hammond TR, Kamitaki N, et al Schizophrenia risk from complex variation of complement component 4. Nature. 2016;530(7589):177–83. 10.1038/nature16549 PMCID: PMC4752392. 26814963 PMC4752392

[50] Blanch A, Roche O, López-Granados E, Fontán G, López-Trascasa M. Detection of C1 inhibitor (SERPING1/C1NH) mutations in exon 8 in patients with hereditary angioedema: evidence for 10 novel mutations. Human Mutation. 2002;20(5):405–6. 10.1002/humu.9073 12402344

[51] Fouche AS, Saunders EFH, Craig T. Depression and anxiety in patients with hereditary angioedema. Ann Allergy Asthma Immunol. 2014;112(4):371–5. 10.1016/j.anai.2013.05.028 24428960 PMC4211935

[52] Rao AR, Yourshaw M, Christensen B, Nelson SF, Kerner B. Rare deleterious mutations are associated with disease in bipolar disorder families. Mol Psychiatry. 2016 10.1038/mp.2016.181 27725659 PMC5388596

[53] Shinozaki G, Potash JB. New developments in the genetics of bipolar disorder. Curr Psychiatry Rep. 2014;16(11):493 10.1007/s11920-014-0493-5 25194313

[54] Benazzi F. Classifying mood disorders by age-at-onset instead of polarity. Progress in Neuro-Psychopharmacology and Biological Psychiatry. 2009;33(1):86–93. 10.1016/j.pnpbp.2008.10.007 18992784

[55] Marcheco-Teruel B, Parra EJ, Fuentes-Smith E, Salas A, Buttenschon HN, Demontis D, et al Cuba: exploring the history of admixture and the genetic basis of pigmentation using autosomal and uniparental markers. PLoS Genet. 2014;10(7):e1004488 10.1371/journal.pgen.1004488 PMCID: 4109857. 25058410 PMC4109857

[56] Adzhubei IA, Schmidt S, Peshkin L, Ramensky VE, Gerasimova A, Bork P, et al A method and server for predicting damaging missense mutations. Nat Meth. 2010;7(4):248–9. 10.1038/nmeth0410-248 20354512 PMC2855889

[57] Chun S, Fay JC. Identification of deleterious mutations within three human genomes. Genome Res. 2009;19(9):1553–61. 10.1101/gr.092619.109 19602639 PMC2752137

[58] Kumar P, Henikoff S, Ng PC. Predicting the effects of coding non-synonymous variants on protein function using the SIFT algorithm. Nat Protoc. 2009;4(7):1073–81. 10.1038/nprot.2009.86 19561590

