# Supplementary figures and images for: Exome sequencing in large, multiplex bipolar disorder families from Cuba
Source: PLoS One. 2018 Oct 31;13(10):e0205895. doi: 10.1371/journal.pone.0205895 (PMC6209204; doi:10.1371/journal.pone.0205895)

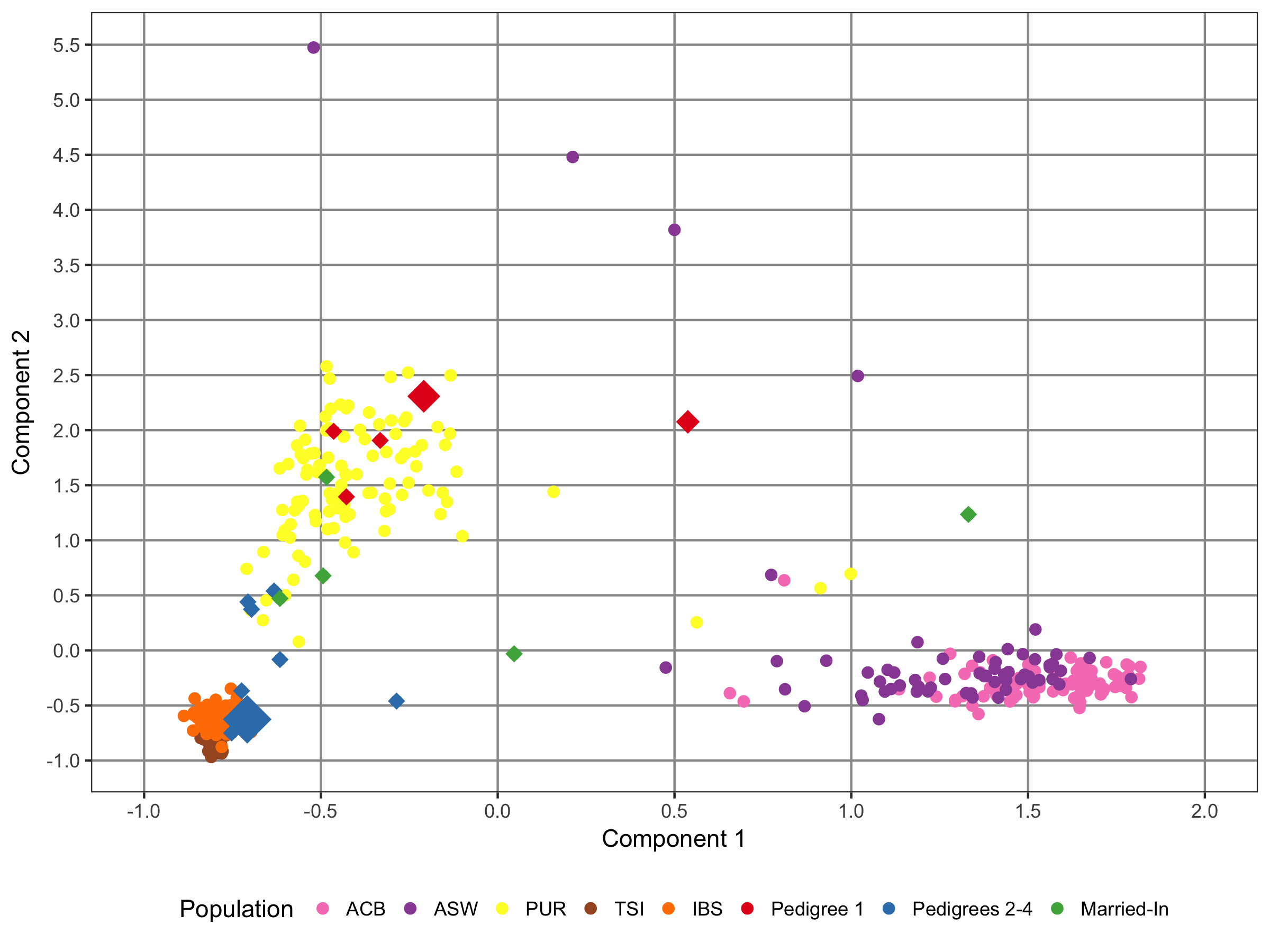

Supplement: S1 Fig — Multidimensional scaling (MDS) components 1 and 2 are displayed. The axes have been scaled to show standard deviations. Red diamonds indicate individuals from pedigree 1. Blue diamonds indicate individuals from subpedigrees 2, 3, and 4. Green diamonds indicate individuals related by marriage only. Diamond size is scaled to indicate the number of subjects represented (n = 1–9 per diamond). 1000 Genomes Project population codes: ACB, African Caribbeans in Barbados (pink); ASW, Americans of African Ancestry in Southwest USA (purple); PUR, Puerto Ricans from Puerto Rico (yellow); TSI, Toscani in Italia (brown); IBS, Iberian Population in Spain (orange). (TIF) [file pone.0205895.s004.tif]

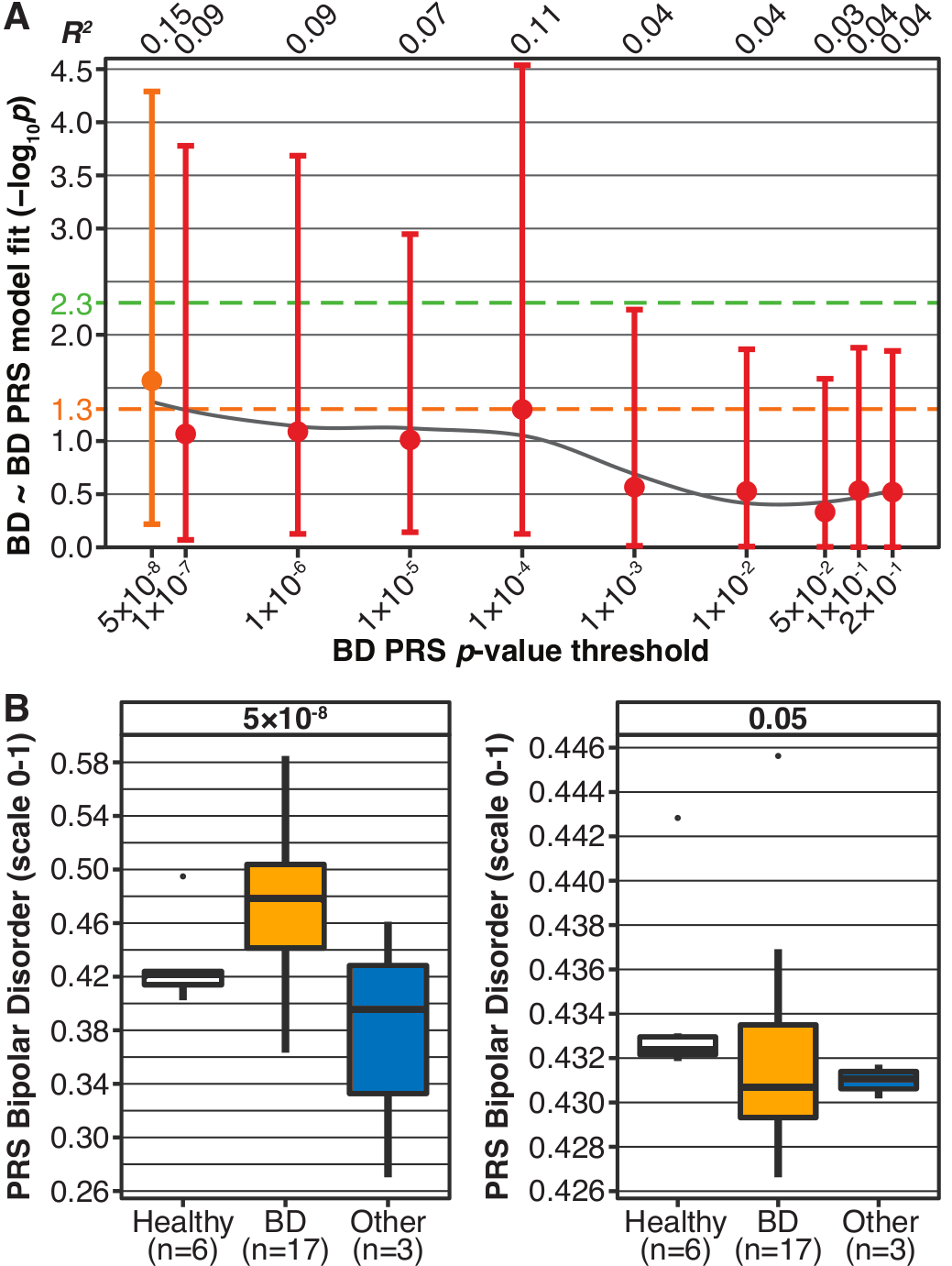

Supplement: S2 Fig — (A) Graphical depiction of test statistics for the association of BD PRS with BD diagnosis, as shown in S2 Table. Association strength is illustrated via p-values on a negative-logarithmic scale including percentile-based 95% bootstrap confidence intervals (CI) for each of the ten PRS based on different p-value thresholds. Nominal significance is indicated by an orange line (-log10(p) = 1.3), and the significance threshold after Bonferroni-correction for ten tests is indicated by a green line (-log10(p) = 2.3). The respective coefficient of determination R2 is shown at the top of the plot. (B) Boxplots of scaled BD PRS for two p-value thresholds, 5×10^−8^ and 0.05. White box represents healthy individuals, orange box represents BD cases, and blue box represents other psychiatric phenotypes. (TIF) [file pone.0205895.s005.tif]
