# Supplementary material for: Exome sequencing in large, multiplex bipolar disorder families from Cuba
Source: PLoS One. 2018 Oct 31;13(10):e0205895. doi: 10.1371/journal.pone.0205895 (PMC6209204; doi:10.1371/journal.pone.0205895)
Supplement: S1 Text — (DOCX) [file pone.0205895.s006.docx]

**Bipolar Disorder Working Group of the Psychiatric Genomics Consortium**

Eli A Stahl1,2,3†&, Gerome Breen4,5†, Andreas J Forstner6,7,8,9,10†, Andrew McQuillin11†, Stephan Ripke12,13,14†, Vassily Trubetskoy13, Manuel Mattheisen15,16,17,18,19, Yunpeng Wang20,21, Jonathan R I Coleman4,5, Héléna A Gaspar4,5, Christiaan A de Leeuw22, Stacy Steinberg23, Jennifer M Whitehead Pavlides24, Maciej Trzaskowski25, Tune H Pers3,26, Peter A Holmans27, Alexander L Richards27, Liam Abbott12, Esben Agerbo19,28,29, Huda Akil30, Diego Albani31, Ney Alliey-Rodriguez32, Thomas D Als15,16,19, Adebayo Anjorin33, Verneri Antilla14, Swapnil Awasthi13, Judith A Badner34, Marie Bækvad-Hansen19,35, Jack D Barchas36, Nicholas Bass11, Michael Bauer37, Richard Belliveau12, Sarah E Bergen38, Carsten Bøcker Pedersen19,28,29, Erlend Bøen39, Marco Boks40, James Boocock41, Monika Budde42, William Bunney43, Margit Burmeister44, Jonas Bybjerg-Grauholm19,35, William Byerley45, Miquel Casas46,47,48,49, Felecia Cerrato12, Pablo Cervantes50, Kimberly Chambert12, Alexander W Charney2, Danfeng Chen12, Claire Churchhouse12,14, Toni-Kim Clarke51, William Coryell52, David W Craig53, Cristiana Cruceanu50,54, David Curtis55,56, Piotr M Czerski57, Anders M Dale58,59,60,61, Simone de Jong4,5, Franziska Degenhardt8,9, Jurgen Del-Favero62, J Raymond DePaulo63, Srdjan Djurovic64,65, Amanda L Dobbyn1,2, Ashley Dumont12, Torbjørn Elvsåshagen66,67, Valentina Escott-Price27, Chun Chieh Fan61, Sascha B Fischer6,10, Matthew Flickinger68, Tatiana M Foroud69, Liz Forty27, Josef Frank70, Christine Fraser27, Nelson B Freimer71, Louise Frisén72,73,74, Katrin Gade42,75, Diane Gage12, Julie Garnham76, Claudia Giambartolomei41, Marianne Giørtz Pedersen19,28,29, Jaqueline Goldstein12, Scott D Gordon77, Katherine Gordon-Smith78, Elaine K Green79, Melissa J Green80, Tiffany A Greenwood60, Jakob Grove15,16,19,81, Weihua Guan82, José Guzman Parra83, Marian L Hamshere27, Martin Hautzinger84, Urs Heilbronner42, Stefan Herms6,8,9,10, Maria Hipolito85, Per Hoffmann6,8,9,10, Dominic Holland58,86, Laura Huckins1,2, Stéphane Jamain87,88, Jessica S Johnson1,2, Anders Juréus38, Radhika Kandaswamy4, Robert Karlsson38, James L Kennedy89,90,91,92, Sarah Kittel-Schneider93, James A Knowles94,95, Manolis Kogevinas96, Anna C Koller8,9, Ralph Kupka97,98,99, Catharina Lavebratt72, Jacob Lawrence100, William B Lawson85, Markus Leber101, Phil H Lee12,14,102, Shawn E Levy103, Jun Z Li104, Chunyu Liu105, Susanne Lucae106, Anna Maaser8,9, Donald J MacIntyre107,108, Pamela B Mahon63,109, Wolfgang Maier110, Lina Martinsson73, Steve McCarroll12,111, Peter McGuffin4, Melvin G McInnis112, James D McKay113, Helena Medeiros95, Sarah E Medland77, Fan Meng30,112, Lili Milani114, Grant W Montgomery25, Derek W Morris115,116, Thomas W Mühleisen6,117, Niamh Mullins4, Hoang Nguyen1,2, Caroline M Nievergelt60,118, Annelie Nordin Adolfsson119, Evaristus A Nwulia85, Claire O'Donovan76, Loes M Olde Loohuis71, Anil P S Ori71, Lilijana Oruc120, Urban Ösby121, Roy H Perlis122,123, Amy Perry78, Andrea Pfennig37, James B Potash63, Shaun M Purcell2,109, Eline J Regeer124, Andreas Reif93, Céline S Reinbold6,10, John P Rice125, Fabio Rivas83, Margarita Rivera4,126, Panos Roussos1,2,127, Douglas M Ruderfer128, Euijung Ryu129, Cristina Sánchez-Mora46,47,49, Alan F Schatzberg130, William A Scheftner131, Nicholas J Schork132, Cynthia Shannon Weickert80,133, Tatyana Shehktman60, Paul D Shilling60, Engilbert Sigurdsson134, Claire Slaney76, Olav B Smeland58,135,136, Janet L Sobell137, Christine Søholm Hansen19,35, Anne T Spijker138, David St Clair139, Michael Steffens140, John S Strauss91,141, Fabian Streit70, Jana Strohmaier70, Szabolcs Szelinger142, Robert C Thompson112, Thorgeir E Thorgeirsson23, Jens Treutlein70, Helmut Vedder143, Weiqing Wang1,2, Stanley J Watson112, Thomas W Weickert80,133, Stephanie H Witt70, Simon Xi144, Wei Xu145,146, Allan H Young147, Peter Zandi148, Peng Zhang149, Sebastian Zollner112, Rolf Adolfsson119, Ingrid Agartz17,39,150, Martin Alda76,151, Lena Backlund73, Bernhard T Baune152, Frank Bellivier153,154,155,156, Wade H Berrettini157, Joanna M Biernacka129, Douglas H R Blackwood51, Michael Boehnke68, Anders D Børglum15,16,19, Aiden Corvin116, Nicholas Craddock27, Mark J Daly12,14, Udo Dannlowski158, Tõnu Esko3,111,114,159, Bruno Etain153,155,156,160, Mark Frye161, Janice M Fullerton133,162, Elliot S Gershon32,163, Michael Gill116, Fernando Goes63, Maria Grigoroiu-Serbanescu164, Joanna Hauser57, David M Hougaard19,35, Christina M Hultman38, Ian Jones27, Lisa A Jones78, René S Kahn2,40, George Kirov27, Mikael Landén38,165, Marion Leboyer88,153,166, Cathryn M Lewis4,5,167, Qingqin S Li168, Jolanta Lissowska169, Nicholas G Martin77,170, Fermin Mayoral83, Susan L McElroy171, Andrew M McIntosh51,172, Francis J McMahon173, Ingrid Melle174,175, Andres Metspalu114,176, Philip B Mitchell80, Gunnar Morken177,178, Ole Mors19,179, Preben Bo Mortensen15,19,28,29, Bertram Müller-Myhsok54,180,181, Richard M Myers103, Benjamin M Neale3,12,14, Vishwajit Nimgaonkar182, Merete Nordentoft19,183, Markus M Nöthen8,9, Michael C O'Donovan27, Ketil J Oedegaard184,185, Michael J Owen27, Sara A Paciga186, Carlos Pato95,187, Michele T Pato95, Danielle Posthuma22,188, Josep Antoni Ramos-Quiroga46,47,48,49, Marta Ribasés46,47,49, Marcella Rietschel70, Guy A Rouleau189,190, Martin Schalling72, Peter R Schofield133,162, Thomas G Schulze42,63,70,75,173, Alessandro Serretti191, Jordan W Smoller12,192,193, Hreinn Stefansson23, Kari Stefansson23,194, Eystein Stordal195,196, Patrick F Sullivan38,197,198, Gustavo Turecki199, Arne E Vaaler200, Eduard Vieta201, John B Vincent141, Thomas Werge19,202,203, John I Nurnberger204, Naomi R Wray24,25, Arianna Di Florio27,198, Howard J Edenberg205, Sven Cichon6,8,10,117, Roel A Ophoff40,41,71, Laura J Scott68, Ole A Andreassen135,136, John Kelsoe60*&, Pamela Sklar1,2*^

† Equal contribution * Co-last authors

^ deceased

**Affiliations:**

1 Department of Genetics and Genomic Sciences, Icahn School of Medicine at Mount Sinai, New York, NY, US

2 Department of Psychiatry, Icahn School of Medicine at Mount Sinai, New York, NY, US

3 Medical and Population Genetics, Broad Institute, Cambridge, MA, US

4 MRC Social, Genetic and Developmental Psychiatry Centre, King's College London, London, GB

5 NIHR BRC for Mental Health, King's College London, London, GB

6 Department of Biomedicine, University of Basel, Basel, CH

7 Department of Psychiatry (UPK), University of Basel, Basel, CH

8 Institute of Human Genetics, University of Bonn School of Medicine & University Hospital Bonn, Bonn, DE

9 Department of Genomics, Life&Brain Center, University of Bonn, Bonn, DE

10 Institute of Medical Genetics and Pathology, University Hospital Basel, Basel, CH

11 Division of Psychiatry, University College London, London, GB

12 Stanley Center for Psychiatric Research, Broad Institute, Cambridge, MA, US

13 Department of Psychiatry and Psychotherapy, Charité - Universitätsmedizin, Berlin, DE

14 Analytic and Translational Genetics Unit, Massachusetts General Hospital, Boston, MA, US

15 iSEQ, Center for Integrative Sequencing, Aarhus University, Aarhus, DK

16 Department of Biomedicine - Human Genetics, Aarhus University, Aarhus, DK

17 Department of Clinical Neuroscience, Centre for Psychiatry Research, Karolinska Institutet, Stockholm, SE

18 Department of Psychiatry, Psychosomatics and Psychotherapy, Center of Mental Health, University Hospital Würzburg, Würzburg, DE

19 iPSYCH, The Lundbeck Foundation Initiative for Integrative Psychiatric Research, DK

20 Institute of Biological Psychiatry, Mental Health Centre Sct. Hans, Copenhagen, DK

21 Institute of Clinical Medicine, University of Oslo, Oslo, NO

22 Department of Complex Trait Genetics, Center for Neurogenomics and Cognitive Research, Amsterdam Neuroscience, Vrije Universiteit Amsterdam, Amsterdam, NL

23 deCODE Genetics / Amgen, Reykjavik, IS

24 Queensland Brain Institute, The University of Queensland, Brisbane, QLD, AU

25 Institute for Molecular Bioscience, The University of Queensland, Brisbane, QLD, AU

26 Division of Endocrinology and Center for Basic and Translational Obesity Research, Boston Children’s Hospital, Boston, MA, US

27 Medical Research Council Centre for Neuropsychiatric Genetics and Genomics, Division of Psychological Medicine and Clinical Neurosciences, Cardiff University, Cardiff, GB

28 National Centre for Register-Based Research, Aarhus University, Aarhus, DK

29 Centre for Integrated Register-based Research, Aarhus University, Aarhus, DK

30 Molecular & Behavioral Neuroscience Institute, University of Michigan, Ann Arbor, MI, US

31 NEUROSCIENCE, Istituto Di Ricerche Farmacologiche Mario Negri, Milano, IT

32 Department of Psychiatry and Behavioral Neuroscience, University of Chicago, Chicago, IL, US

33 Psychiatry, Berkshire Healthcare NHS Foundation Trust, Bracknell, GB

34 Psychiatry, Rush University Medical Center, Chicago, IL, US

35 Center for Neonatal Screening, Department for Congenital Disorders, Statens Serum Institut, Copenhagen, DK

36 Department of Psychiatry, Weill Cornell Medical College, New York, NY, US

37 Department of Psychiatry and Psychotherapy, University Hospital Carl Gustav Carus, Technische Universität Dresden, Dresden, DE

38 Department of Medical Epidemiology and Biostatistics, Karolinska Institutet, Stockholm, SE

39 Department of Psychiatric Research, Diakonhjemmet Hospital, Oslo, NO

40 Psychiatry, UMC Utrecht Hersencentrum Rudolf Magnus, Utrecht, NL

41 Human Genetics, University of California Los Angeles, Los Angeles, CA, US

42 Institute of Psychiatric Phenomics and Genomics (IPPG), University Hospital, LMU Munich, Munich, DE

43 Department of Psychiatry and Human Behavior, University of California, Irvine, Irvine, CA, US

44 Molecular & Behavioral Neuroscience Institute and Department of Computational Medicine & Bioinformatics, University of Michigan, Ann Arbor, MI, US

45 Psychiatry, University of California San Francisco, San Francisco, CA, US

46 Instituto de Salud Carlos III, Biomedical Network Research Centre on Mental Health (CIBERSAM), Madrid, ES

47 Department of Psychiatry, Hospital Universitari Vall d´Hebron, Barcelona, ES

48 Department of Psychiatry and Forensic Medicine, Universitat Autònoma de Barcelona, Barcelona, ES

49 Psychiatric Genetics Unit, Group of Psychiatry Mental Health and Addictions, Vall d´Hebron Research Institut (VHIR), Universitat Autònoma de Barcelona, Barcelona, ES

50 Department of Psychiatry, Mood Disorders Program, McGill University Health Center, Montreal, QC, CA

51 Division of Psychiatry, University of Edinburgh, Edinburgh, GB

52 University of Iowa Hospitals and Clinics, Iowa City, IA, US

53 Translational Genomics, USC, Phoenix, AZ, US

54 Department of Translational Research in Psychiatry, Max Planck Institute of Psychiatry, Munich, DE

55 Centre for Psychiatry, Queen Mary University of London, London, GB

56 UCL Genetics Institute, University College London, London, GB

57 Department of Psychiatry, Laboratory of Psychiatric Genetics, Poznan University of Medical Sciences, Poznan, PL

58 Department of Neurosciences, University of California San Diego, La Jolla, CA, US

59 Department of Radiology, University of California San Diego, La Jolla, CA, US

60 Department of Psychiatry, University of California San Diego, La Jolla, CA, US

61 Department of Cognitive Science, University of California San Diego, La Jolla, CA, US

62 Applied Molecular Genomics Unit, VIB Department of Molecular Genetics, University of Antwerp, Antwerp, Belgium

63 Department of Psychiatry and Behavioral Sciences, Johns Hopkins University School of Medicine, Baltimore, MD, US

64 Department of Medical Genetics, Oslo University Hospital Ullevål, Oslo, NO

65 NORMENT, KG Jebsen Centre for Psychosis Research, Department of Clinical Science, University of Bergen, Bergen, NO

66 Department of Neurology, Oslo University Hospital, Oslo, NO

67 NORMENT, KG Jebsen Centre for Psychosis Research, Oslo University Hospital, Oslo, NO

68 Center for Statistical Genetics and Department of Biostatistics, University of Michigan, Ann Arbor, MI, US

69 Department of Medical & Molecular Genetics, Indiana University, Indianapolis, IN, US

70 Department of Genetic Epidemiology in Psychiatry, Central Institute of Mental Health, Medical Faculty Mannheim, Heidelberg University, Mannheim, DE

71 Center for Neurobehavioral Genetics, University of California Los Angeles, Los Angeles, CA, US

72 Department of Molecular Medicine and Surgery, Karolinska Institutet and Center for Molecular Medicine, Karolinska University Hospital, Stockholm, SE

73 Department of Clinical Neuroscience, Karolinska Institutet and Center for Molecular Medicine, Karolinska University Hospital, Stockholm, SE

74 Child and Adolescent Psychiatry Research Center, Stockholm, SE

75 Department of Psychiatry and Psychotherapy, University Medical Center Göttingen, Göttingen, DE

76 Department of Psychiatry, Dalhousie University, Halifax, NS, CA

77 Genetics and Computational Biology, QIMR Berghofer Medical Research Institute, Brisbane, QLD, AU

78 Department of Psychological Medicine, University of Worcester, Worcester, GB

79 School of Biomedical and Healthcare Sciences, Plymouth University Peninsula Schools of Medicine and Dentistry, Plymouth, GB

80 School of Psychiatry, University of New South Wales, Sydney, NSW, AU

81 Bioinformatics Research Centre, Aarhus University, Aarhus, DK

82 Biostatistics, University of Minnesota System, Minneapolis, MN, US

83 Mental Health Department, University Regional Hospital, Biomedicine Institute (IBIMA), Málaga, ES

84 Department of Psychology, Eberhard Karls Universität Tübingen, Tubingen, DE

85 Department of Psychiatry and Behavioral Sciences, Howard University Hospital, Washington, DC, US

86 Center for Multimodal Imaging and Genetics, University of California San Diego, La Jolla, CA, US

87 Psychiatrie Translationnelle, Inserm U955, Créteil, FR

88 Faculté de Médecine, Université Paris Est, Créteil, FR

89 Campbell Family Mental Health Research Institute, Centre for Addiction and Mental Health, Toronto, ON, CA

90 Neurogenetics Section, Centre for Addiction and Mental Health, Toronto, ON, CA

91 Department of Psychiatry, University of Toronto, Toronto, ON, CA

92 Institute of Medical Sciences, University of Toronto, Toronto, ON, CA

93 Department of Psychiatry, Psychosomatic Medicine and Psychotherapy, University Hospital Frankfurt, Frankfurt am Main, DE

94 Cell Biology, SUNY Downstate Medical Center College of Medicine, Brooklyn, NY, US

95 Institute for Genomic Health, SUNY Downstate Medical Center College of Medicine, Brooklyn, NY, US

96 ISGlobal, Barcelona, ES

97 Psychiatry, Altrecht, Utrecht, NL

98 Psychiatry, GGZ inGeest, Amsterdam, NL

99 Psychiatry, VU medisch centrum, Amsterdam, NL

100 Psychiatry, North East London NHS Foundation Trust, Ilford, GB

101 Clinic for Psychiatry and Psychotherapy, University Hospital Cologne, Cologne, DE

102 Psychiatric and Neurodevelopmental Genetics Unit, Massachusetts General Hospital, Boston, MA, US

103 HudsonAlpha Institute for Biotechnology, Huntsville, AL, US

104 Department of Human Genetics, University of Michigan, Ann Arbor, MI, US

105 Psychiatry, University of Illinois at Chicago College of Medicine, Chicago, IL, US

106 Max Planck Institute of Psychiatry, Munich, DE

107 Mental Health, NHS 24, Glasgow, GB

108 Division of Psychiatry, Centre for Clinical Brain Sciences, University of Edinburgh, Edinburgh, GB

109 Psychiatry, Brigham and Women's Hospital, Boston, MA, US

110 Department of Psychiatry and Psychotherapy, University of Bonn, Bonn, DE

111 Department of Genetics, Harvard Medical School, Boston, MA, US

112 Department of Psychiatry, University of Michigan, Ann Arbor, MI, US

113 Genetic Cancer Susceptibility Group, International Agency for Research on Cancer, Lyon, FR

114 Estonian Genome Center, University of Tartu, Tartu, EE

115 Discipline of Biochemistry, Neuroimaging and Cognitive Genomics (NICOG) Centre, National University of Ireland, Galway, Galway, IE

116 Neuropsychiatric Genetics Research Group, Dept of Psychiatry and Trinity Translational Medicine Institute, Trinity College Dublin, Dublin, IE

117 Institute of Neuroscience and Medicine (INM-1), Research Centre Jülich, Jülich, DE

118 Research/Psychiatry, Veterans Affairs San Diego Healthcare System, San Diego, CA, US

119 Department of Clinical Sciences, Psychiatry, Umeå University Medical Faculty, Umeå, SE

120 Department of Clinical Psychiatry, Psychiatry Clinic, Clinical Center University of Sarajevo, Sarajevo, BA

121 Department of Neurobiology, Care sciences, and Society, Karolinska Institutet and Center for Molecular Medicine, Karolinska University Hospital, Stockholm, SE

122 Psychiatry, Harvard Medical School, Boston, MA, US

123 Division of Clinical Research, Massachusetts General Hospital, Boston, MA, US

124 Outpatient Clinic for Bipolar Disorder, Altrecht, Utrecht, NL

125 Department of Psychiatry, Washington University in Saint Louis, Saint Louis, MO, US

126 Department of Biochemistry and Molecular Biology II, Institute of Neurosciences, Center for Biomedical Research, University of Granada, Granada, ES

127 Department of Neuroscience, Icahn School of Medicine at Mount Sinai, New York, NY, US

128 Medicine, Psychiatry, Biomedical Informatics, Vanderbilt University Medical Center, Nashville, TN, US

129 Department of Health Sciences Research, Mayo Clinic, Rochester, MN, US

130 Psychiatry and Behavioral Sciences, Stanford University School of Medicine, Stanford, CA, US

131 Rush University Medical Center, Chicago, IL, US

132 Scripps Translational Science Institute, La Jolla, CA, US

133 Neuroscience Research Australia, Sydney, NSW, AU

134 Faculty of Medicine, Department of Psychiatry, School of Health Sciences, University of Iceland, Reykjavik, IS

135 Div Mental Health and Addiction, Oslo University Hospital, Oslo, NO

136 NORMENT, University of Oslo, Oslo, NO

137 Psychiatry and the Behavioral Sciences, University of Southern California, Los Angeles, CA, US

138 Mood Disorders, PsyQ, Rotterdam, NL

139 Institute for Medical Sciences, University of Aberdeen, Aberdeen, UK

140 Research Division, Federal Institute for Drugs and Medical Devices (BfArM), Bonn, DE

141 Centre for Addiction and Mental Health, Toronto, ON, CA

142 Neurogenomics, TGen, Los Angeles, AZ, US

143 Psychiatry, Psychiatrisches Zentrum Nordbaden, Wiesloch, DE

144 Computational Sciences Center of Emphasis, Pfizer Global Research and Development, Cambridge, MA, US

145 Department of Biostatistics, Princess Margaret Cancer Centre, Toronto, ON, CA

146 Dalla Lana School of Public Health, University of Toronto, Toronto, ON, CA

147 Psychological Medicine, Institute of Psychiatry, Psychology & Neuroscience, King's College London, London, GB

148 Department of Mental Health, Johns Hopkins University Bloomberg School of Public Health, Baltimore, MD, US

149 Institute of Genetic Medicine, Johns Hopkins University School of Medicine, Baltimore, MD, US

150 NORMENT, KG Jebsen Centre for Psychosis Research, Division of Mental Health and Addiction, Institute of Clinical Medicine and Diakonhjemmet Hospital, University of Oslo, Oslo, NO

151 National Institute of Mental Health, Klecany, CZ

152 Discipline of Psychiatry, University of Adelaide, Adelaide, SA, AU

153 Department of Psychiatry and Addiction Medicine, Assistance Publique - Hôpitaux de Paris, Paris, FR

154 Paris Bipolar and TRD Expert Centres, FondaMental Foundation, Paris, FR

155 UMR-S1144 Team 1: Biomarkers of relapse and therapeutic response in addiction and mood disorders, INSERM, Paris, FR

156 Psychiatry, Université Paris Diderot, Paris, FR

157 Psychiatry, University of Pennsylvania, Philadelphia, PA, US

158 Department of Psychiatry, University of Münster, Münster, DE

159 Division of Endocrinology, Children's Hospital Boston, Boston, MA, US

160 Centre for Affective Disorders, Institute of Psychiatry, Psychology and Neuroscience, London, GB

161 Department of Psychiatry & Psychology, Mayo Clinic, Rochester, MN, US

162 School of Medical Sciences, University of New South Wales, Sydney, NSW, AU

163 Department of Human Genetics, University of Chicago, Chicago, IL, US

164 Biometric Psychiatric Genetics Research Unit, Alexandru Obregia Clinical Psychiatric Hospital, Bucharest, RO

165 Institute of Neuroscience and Physiology, University of Gothenburg, Gothenburg, SE

166 INSERM, Paris, FR

167 Department of Medical & Molecular Genetics, King's College London, London, GB

168 Neuroscience Therapeutic Area, Janssen Research and Development, LLC, Titusville, NJ, US

169 Cancer Epidemiology and Prevention, M. Sklodowska-Curie Cancer Center and Institute of Oncology, Warsaw, PL

170 School of Psychology, The University of Queensland, Brisbane, QLD, AU

171 Research Institute, Lindner Center of HOPE, Mason, OH, US

172 Centre for Cognitive Ageing and Cognitive Epidemiology, University of Edinburgh, Edinburgh, GB

173 Human Genetics Branch, Intramural Research Program, National Institute of Mental Health, Bethesda, MD, US

174 Division of Mental Health and Addiction, Oslo University Hospital, Oslo, NO

175 Division of Mental Health and Addiction, University of Oslo, Institute of Clinical Medicine, Oslo, NO

176 Institute of Molecular and Cell Biology, University of Tartu, Tartu, EE

177 Mental Health, Faculty of Medicine and Health Sciences, Norwegian University of Science and Technology - NTNU, Trondheim, NO

178 Psychiatry, St Olavs University Hospital, Trondheim, NO

179 Psychosis Research Unit, Aarhus University Hospital, Risskov, DK

180 Munich Cluster for Systems Neurology (SyNergy), Munich, DE

181 University of Liverpool, Liverpool, GB

182 Psychiatry and Human Genetics, University of Pittsburgh, Pittsburgh, PA, US

183 Mental Health Services in the Capital Region of Denmark, Mental Health Center Copenhagen, University of Copenhagen, Copenhagen, DK

184 Division of Psychiatry, Haukeland Universitetssjukehus, Bergen, NO

185 Faculty of Medicine and Dentistry, University of Bergen, Bergen, NO

186 Human Genetics and Computational Biomedicine, Pfizer Global Research and Development, Groton, CT, US

187 College of Medicine Institute for Genomic Health, SUNY Downstate Medical Center College of Medicine, Brooklyn, NY, US

188 Department of Clinical Genetics, Amsterdam Neuroscience, Vrije Universiteit Medical Center, Amsterdam, NL

189 Department of Neurology and Neurosurgery, McGill University, Faculty of Medicine, Montreal, QC, CA

190 Montreal Neurological Institute and Hospital, Montreal, QC, CA

191 Department of Biomedical and NeuroMotor Sciences, University of Bologna, Bologna, IT

192 Department of Psychiatry, Massachusetts General Hospital, Boston, MA, US

193 Psychiatric and Neurodevelopmental Genetics Unit (PNGU), Massachusetts General Hospital, Boston, MA, US

194 Faculty of Medicine, University of Iceland, Reykjavik, IS

195 Department of Psychiatry, Hospital Namsos, Namsos, NO

196 Department of Neuroscience, Norges Teknisk Naturvitenskapelige Universitet Fakultet for naturvitenskap og teknologi, Trondheim, NO

197 Department of Genetics, University of North Carolina at Chapel Hill, Chapel Hill, NC, US

198 Department of Psychiatry, University of North Carolina at Chapel Hill, Chapel Hill, NC, US

199 Department of Psychiatry, McGill University, Montreal, QC, CA

200 Dept of Psychiatry, Sankt Olavs Hospital Universitetssykehuset i Trondheim, Trondheim, NO

201 Clinical Institute of Neuroscience, Hospital Clinic, University of Barcelona, IDIBAPS, CIBERSAM, Barcelona, ES

202 Institute of Biological Psychiatry, MHC Sct. Hans, Mental Health Services Copenhagen, Roskilde, DK

203 Department of Clinical Medicine, University of Copenhagen, Copenhagen, DK

204 Psychiatry, Indiana University School of Medicine, Indianapolis, IN, US

205 Biochemistry and Molecular Biology, Indiana University School of Medicine, Indianapolis, IN, US
